# Where did I leave my systematic review protocol, and what should it contain regarding Trial Sequential Analysis?

**DOI:** 10.1186/s12874-026-02775-7

**Published:** 2026-01-29

**Authors:** Mark Aninakwah Asante, Vibeke Wagner, Sigurlaug Hanna Hafliðadóttir, Buddheera WMB Kumburegama, Elisabeth Buck Pedersen, Johanne Pereira Ribeiro, Julie Perrine Schaug, Joachim Birch Milan, Markus Harboe Olsen, Christina Madsen, Christian Gunge Riberholt, Christian Gluud

**Affiliations:** 1 Copenhagen Trial Unit, Centre for Clinical Intervention Research, The Capital Region, Copenhagen University Hospital − Rigshospitalet, Blegdamsvej 9, Copenhagen, 2100 Denmark; 2 Department of Brain and Spinal Cord Injury, Neuroscience Centre, Copenhagen University Hospital − Rigshospitalet, Valdemarsvej 23, Glostrup, 2600 Denmark; 3 Bjarg Rehabilitation Center, Bugðusíðu 1, Akureyri, 603 Iceland; 4 Center for Evidence-Based Psychiatry, Psychiatric Research Unit, Psychiatry Region Zealand, Fælledvej 6, Slagelse, Region Zealand 4200 Denmark; 5 Division of Psychiatry, Department of Clinical Sciences, Malmö, Faculty of Medicine, Lund University, Lund, Sweden; 6 Department of Neuroanaesthesiology, Neuroscience Centre, Copenhagen University Hospital − Rigshospitalet, Blegdamsvej 9, Copenhagen, 2100 Denmark; 7 Department of Clinical Medicine, Faculty of Health and Medical Sciences, University of Copenhagen, Blegdamsvej 3B, Copenhagen, Denmark; 8 Department of Regional Health Research, The Faculty of Health Sciences, University of Southern Denmark, Odense, Denmark

**Keywords:** Meta-analysis, Methodology, Systematic review, Protocol, Trial sequential analysis, Continuous outcomes, Dichotomous outcomes

## Abstract

**Background:**

Publishing a protocol before starting a systematic review is necessary to ensure data integrity and trustworthiness of results. Trial Sequential Analysis (TSA) is a program and analysis method to control random type I and type II errors often used in systematic reviews (having a protocol in some form) and meta-analyses publications (having no protocol). We scrutinised protocols for systematic reviews and meta-analyses reports to examine their quality and transparency focusing on TSA parameters.

**Methods:**

We searched Medline and the Cochrane Database of Systematic Reviews from 1 January 2018 to 31 December, 2021 for published systematic reviews and meta-analysis reports that included a TSA. We included reports that had at least two randomised clinical trials analysed in a forest plot plus in a TSA. Two independent researchers extracted data to assess coherence between the protocol and its systematic review. Disagreements were resolved through discussion or arbitration.

**Results:**

We included 270 systematic reviews with pre-published protocols and 274 meta-analyses without protocols. Among the systematic reviews, 135 (50%) planned to perform TSA in their protocols (112 dichotomous and 23 continuous outcomes). TSA parameters between protocols and published reviews were often inconsistent. For dichotomous outcomes, consistency was highest for alpha (50.9%) and lowest for power (1.8%). For continuous outcomes, consistency was minimal across all parameters. Statistically significant deviations between planned and actual TSA values were observed for statistical power (mean increase 8.95%; 95% CI 7.43% to 10.47%; *p* < 0.001) and heterogeneity (43.8% vs. 57.1%; *p* = 0.048) in dichotomous outcomes. Systematic reviews with predefined TSA parameters in protocols demonstrated significantly better reporting of the proportion of participants with the outcome in the control groups, alpha, and heterogeneity compared to those without protocol information. These patterns persisted when including the meta-analyses reports. Systematic reviews with continuous outcomes showed similar trends, though differences were not statistically significant.

**Conclusion:**

TSA is often neglected in systematic reviews protocols and when planned the TSA parameters are inconsistently reported and followed. Predefined protocols improve transparency and reporting, and should be required to enhance reproducibility, reduce bias, and prevent research waste in systematic reviews.

## Introduction

Systematic reviews of randomised clinical trials are considered the top of the evidence hierarchy. The common goal of these publications is to produce dependable recommendations for prevention, diagnosis, prognosis, treatment, and care of the best available evidence [1, 2]. A PubMed search conducted on 11 August, 2025 showed that in 2024 alone, over 38,000 meta-analyses and 53,000 systematic reviews were published. Without rigorous methods especially in planning and applying Trial Sequential Analysis (TSA), their conclusions risk misleading rather than guiding clinical decisions.

The protocol of a systematic review is the blueprint of the systematic review, stipulating the objectives, outcomes, and methods of interest of the systematic review. This enhances transparency and is helpful in the management of the review. Preparing a protocol before conducting a systematic review is essential to ensure that all methodological decisions, from the search terms to the data extraction and synthesis process, are thoroughly considered and justified, thus increasing data integrity and trustworthiness [1, 3, 4]. Thus, a well-developed protocol is likely to produce reliable evidence. This is helpful in the management of the review and enhances transparency [3]. Meta-analyses should also rely on pre-specified protocols to maintain methodological rigor. However, most if not all meta-analyses reports are conducted without a publicly available, pre-registered protocol which undermines their credibility and overall value [2, 5–8].

Both systematic reviews and meta-analysis reports synthesise the results of multiple randomised clinical trials using strategies that limit bias (systematic errors) and play of chance (random errors) [9, 10]. TSA is a frequentist statistical methodology used to control random type I and type II errors [11–13]. Since its introduction in 2005, the TSA method has been utilised in a number of systematic reviews and meta-analyses [14, 15]. TSA can easily be misapplied and misinterpreted if parameters are not predefined before commencing the systematic review. For dichotomous outcomes (Table 1), parameters are proportion of participants with the outcome in the control group, relative risk reduction, alpha level, beta level (and hence power = 1.0 – beta), and heterogeneity; for continuous outcome (Table 2), parameters are minimally relevant clinical difference, variance (or standard deviation), alpha level, beta level (and hence power = 1.0 – beta), and heterogeneity. These parameters should be documented in pre-published or registered protocols [16]. Failure to do so can significantly affect the analytical results and potentially mislead clinical practice [15–18].

**Table 1:** Parameters for dichotomous outcomes in calculation of diversity-adjusted required information size in Trial Sequential Analysis

Parameters for dichotomous outcomes:
Pc: Proportion of participants with the outcomes in the control group
RRR: Relative risk reduction
Alpha: Type I error
Beta: Type II error
D^2^ : Diversity

**Table 2:** Parameters for continuous outcomes in calculation of diversity-adjusted required information size in Trial Sequential Analysis

Parameters for continuous outcomes:
Variance
MIREDIF: Minimal relevant difference
Alpha: Type I error
Beta: Type II error
D^2^ : Diversity

By developing and publishing detailed protocols, research methods can be thoroughly checked and duplication of research can be reduced [6, 19]. van der Braak and colleagues found that only 38% of the so called ‘systematic reviews’ included in their analysis had registered a protocol [20]. Furthermore, Tawfik and colleagues revealed that authors need more information regarding the obligation and importance of registering systematic reviews or meta-analysis protocols in advance [21]. We have previously demonstrated problems with missing protocols and insufficiently reported protocols for systematic reviews [16, 22]. In the present study, we report more detailed on protocol availability and the consistency between the protocol and the systematic review. Moreover, we compare TSA parameters in systematic reviews (all with a protocol) having a TSA protocol to systematic reviews without a TSA protocol and to meta-analysis reports (lacking protocols by definition) published from 1 January, 2018 to 31 December, 2021 [16, 22].

## Methods

This methodological research-on-research review was conducted in compliance with the reporting guidelines outlined in the Preferred Reporting Items for Systematic Reviews and Meta-Analysis (PRISMA) statement [23]. We followed the recommendations outlined in the Cochrane Handbook of Systematic Reviews of Interventions [7]. Our methods are described in detail in our published protocol [22] and in our main Major Mistakes or Errors in the use of Trial Sequential Analysis (METSA) study report [16]. The study protocol was in accordance with PRISMA-P guidelines and was registered with PROSPERO on 18 September 2021 (CRD42021273811).

### Search strategy and data extraction

An experienced information specialist performed the literature searches. For details regarding databases, searches, and screening methods see our published protocol [22]. We searched for systematic reviews and meta-analysis reports published between 1 January 2018 to 31 December 2021 that contained a minimum of two randomised clinical trials and featured at least one meta-analysis plus one TSA. We did not impose any specific requirements for the type of participants, interventions, or outcomes.

Studies with pre-published protocol and/or registration completed before data extraction were considered as systematic reviews, whereas studies lacking a protocol or prior registration were considered as meta-analysis reports. Studies were uploaded together with their protocols into Covidence systematic review software (Veritas Health Innovation, Melbourne, Australia) and were crosschecked before data extraction. Two independent authors extracted the data into REDCap (Research Electronic Data Capture, Vanderbilt University, Nashville, TN, USA) hosted at The Capital Region, Denmark [16, 24]. We worked with international and national researchers other than from the Copenhagen Trial Unit all being new to TSA, hence none of the assessors extracted data from papers they had authored. Discrepancies were solely based on document inspection and consistency on the exact numeric match in situations where changes were not justified in review. Inter-rater agreements were measured by kappa [16]. Systematic review quality, was assessed using the AMSTAR II [25]. For studies where data extraction had started before the protocol was published or the study registration, were considered meta-analysis reports, and quality was not assessed. We extracted data on protocol availability, coherency between protocol and review, and planning of the TSA. Any disparities were resolved by discussion or a third party as an arbitrator [16].

### Statistical analysis

We performed descriptive statistics using R version 4.2.1 (R Core Team, Vienna, Austria). We derived frequencies from our total METSA sample and presented them as counts and percentages [26]. The presence of consistencies and inconsistencies are presented as n and percentages in tables. Paired and unpaired mean differences with 95% confidence intervals were calculated for TSA parameters, as well as relative risks with 95% confidence intervals using log-binomial regression for dichotomous data. Statistical significance was defined as *p* < 0.05.

## Results

We included 544 records consisting of 270 (49%) systematic reviews (with a pre-published protocol and/or registered) and 274 meta-analysis reports (without a pre-published protocol nor registered) all containing a TSA of at least two randomised clinical trials. Of the 270 systematic reviews with a published protocol, 135 (50%) included TSA in their protocol [16]. Most systematic reviews had a publicly available registration in PROSPERO (85%) while 44/544 (16%) had a published protocol in a scientific peer-reviewed journal, including 27 (5%) Cochrane reviews [16]. In our AMSTAR II assessment, twenty-seven (10%) of systematic reviews were rated at high, 18 (7%) at moderate, 35 (13%) at low, and 190 (70%) at critically low confidence in their results [16]. All 274 meta-analysis reports (studies without a protocol) were considered of critically low confidence in their results [16]. The overall agreement between reviewers was calculated on selected items in the data-extraction form and showed from moderate to almost perfect agreement as shown in Supplemental Table 9 of our main METSA study [16].

### Reporting and consistency of TSA parameters between protocol and the systematic review

#### Dichotomous outcomes

Out of 112 systematic reviews with dichotomous outcomes, reporting of TSA parameters was inconsistent. The proportion of participants with the outcome in the control group (Pc) was reported in 82.1% of protocols and 73.2% of the systematic reviews, but only 38.4% showed consistency between the TSA protocol and the systematic review. Similarly, the relative risk reduction (RRR) was reported in 38.4% of protocols and 83.0% of the systematic reviews, with only 32.1% consistency. Alpha levels were specified in 61.6% of protocols and 96.4% of systematic reviews, yet consistency was seen in just 50.9% of cases. Power was rarely consistent, with only 1.8% agreement, despite being reported in approximately half of the protocols and systematic reviews. Heterogeneity assumptions were reported in 43.8% of protocols and 57.1% of systematic reviews, but consistency was limited to 33.0% (Table 3).

**Table 3:** Reporting and consistency between protocol and review regarding TSA parameters used in systematic reviews on dichotomous outcomes

	Dichotomous; *n* = 112
Pc	
Pc in protocol	92 (82.1%)
Pc in review	82 (73.2%)
Pc agreement	43 (38.4%)
Not defined in protocol and/or study	41 (36.6%)
RRR	
RRR in protocol	43 (38.4%)
RRR in review	93 (83.0%)
RRR agreement	36 (32.1%)
Not defined in protocol and/or study	69 (61.6%)
Alpha	
Alpha in protocol	69 (61.6%)
Alpha in review	108 (96.4%)
Alpha agreement	57 (50.9%)
Not defined in protocol and/or study	43 (38.4%)
Power	
Power in protocol	57 (50.9%)
Power in review	61 (54.5%)
Power agreement^*^	2 (1.8%)
Not defined in protocol and/or study	93 (83.0%)
Heterogeneity	
Heterogeneity in protocol	49 (43.8%)
Heterogeneity in review	64 (57.1%)
Heterogeneity agreement	37 (33.0%)
Not defined in protocol and/or study	71 (63.4%)

“Not defined in protocol and/or study” were studies that did not have the parameter mentioned either in protocol nor studies

*Pc* Proportion of participants with outcome in the control group, *RRR* Relative risk reduction, *n* total number of studies

^*^Power parameter showed the lowest consistency

In paired comparisons, the values used in systematic reviews frequently differed from those planned in protocols. Statistically significant differences were observed for power (mean increase 8.95%; 95% CI 7.43% to 10.47%; *p* < 0.001) and heterogeneity assumptions (RR 1.31; 95% CI 1.01 to 1.72; *p* = 0.048). Differences in Pc, RRR, and alpha were not statistically significant (*p* > 0.05) (Table 4).

**Table 4:** TSA parameters planned in the protocol of the systematic review compared with actual TSA parameters values used in the published review across dichotomous outcomes (paired data)

	Sample size (*n*)	Planned mean (95% CI) /Planned reported *n* (%)	Actual mean (95% CI) /Actual reported *n* (%)	Mean difference (95% CI) /Relative Risk (95%CI)	*p*-value
Pc	112	92 (82.1%)	82 (73.2%)	0.89 (0.77 to 1.03)	0.111
RRR	43	0.183 (0.162 to 0.204)	0.201 (0.158 to 0.245)	0.0182 (-0.0154 to 0.0518)	0.280
Alpha	69	0.042 (0.039 to 0.045)	0.041 (0.038 to 0.043)	-0.0011 (-0.0031 to 8e-04)	0.255
Power^a*^	19	0.811 (0.795 to 0.826)	0.900 (0.900 to 0.900)	0.0895 (0.0743 to 0.1047)	< 0.001
Heterogeneity*	112	49 (43.8%)	64 (57.1%)	1.31 (1.01 to 1.72)	0.048

*Pc* Proportion of participants with the outcome in the control group, *RRR* Relative risk reduction, *n* total number of studies

^a^Mean difference in statistical power is reported as a proportion and should be interpreted in percentage points, representing the absolute difference between planned and applied power in the TSA

^*^Power and heterogeneity parameters showed a significant difference

Systematic reviews including TSA parameters in their protocols (*n* = 112) had significantly higher reporting of Pc (73.2% vs. 56.6%; RR 1.29; *p* = 0.012) and heterogeneity (57.1% vs. 36.8%; RR 1.55; *p* = 0.004), compared to those without TSA parameter information. A significant difference was also observed in alpha levels (mean difference 0.004; *p* = 0.002) (Table 5).

**Table 5:** Comparison of TSA parameters used in systematic reviews having planned TSA parameters in their protocol to systematic reviews without detailed information of TSA parameters in their protocol on dichotomous outcomes

	Mean (95% CI) in those with protocol information / those with protocol information *n* (%) *n* = 112	Mean (95% CI) in those without protocol information / those without protocol information *n* (%) *n* = 106	Mean difference (95% CI) / Relative Risk (95% CI)	*p*-value
Pc*	82 (73.2%)	60 (56.6%)	1.29 (1.06 to 1.60)	0.012
RRR	0.222 (0.192 to 0.251)	0.213 (0.187 to 0.239)	0.009 (-0.030 to 0.048)	0.662
Alpha^*^	0.044 (0.042 to 0.046)	0.048 (0.046 to 0.049)	-0.004 (0.007 to 0.001)	0.002
Power^a^	0.900 (0.896 to 0.904)	0.891 (0.875 to 0.908)	-0.009 (-0.008 to 0.025)	0.306
Heterogeneity*	64 (57.1%)	39 (36.8%)	1.55 (1.16 to 2.12)	0.004

*Pc, alpha, and heterogeneity parameters showed a significant difference

*Pc* Proportion of participants with the outcome in the control group, *RRR* Relative risk reduction, *n* total number of studies

^a^Mean difference in statistical power is reported as a proportion and should be interpreted in percentage points, representing the absolute difference between planned and applied power in the TSA

### Continuous outcomes

Among 23 systematic reviews with continuous outcomes, reporting was generally poor. Variance was reported in only 8.7% of protocols and 26.1% of systematic reviews, with no cases of consistency. The minimal relevant difference (MIREDIF) was not specified in any protocol but reported in 30.4% of the systematic reviews. Alpha and power were reported more frequently (43.5% and 30.4% in protocols, respectively; 78.3% and 52.2% in systematic reviews), yet consistency was low (30.4% for alpha and 0.0% for power). Heterogeneity was addressed in 17.4% of protocols and 43.5% of reviews, with 8.7% consistency (Table 6).

**Table 6:** Reporting and consistency between protocol and review regarding TSA parameters in systematic reviews on continuous outcomes

	Continuous; *n* = 23
Variance	
Variance in protocol	2 (8.7%)
Variance in review	6 (26.1%)
Variance not in agreement - Not defined in protocol and/or study	21 (91.3%)
MIREDIF	
MIREDIF in protocol^*^	0 (0.0%)
MIREDIF in review	7 (30.4%)
MIREDIF not in agreement - Not defined in protocol and/or review	23 (100.0%)
Alpha	
Alpha in protocol	10 (43.5%)
Alpha in review	18 (78.3%)
Alpha agreement	7 (30.4%)
Not defined in protocol and/or review	13 (56.5%)
Power	
Power in protocol	7 (30.4%)
Power in review	12 (52.2%)
Power agreement	0 (0.0%)
Not defined in protocol and/or review	21 (91.3%)
Heterogeneity	
Heterogeneity in protocol	4 (17.4%)
Heterogeneity in review	10 (43.5%)
Heterogeneity agreement	2 (8.7%)
Not defined in protocol and/or review	20 (87.0%)

“Not defined in protocol and/or study” were studies that did not have the parameter mentioned either in protocol nor studies

*MIREDIF* Minimal relevant difference, *n* total number of studies

*None of the studies pre-planned on MIREDIF in the protocol

For continuous outcomes, discrepancies between planned and used TSA parameters were also observed. However, none of the comparisons reached statistical significance. Although the reporting of variance and heterogeneity increased in the systematic review phase, the differences in alpha, power, and heterogeneity parameters were not significant (all *p* > 0.05). MIREDIF was neither reported in the protocols nor the systematic reviews (Table 7).

**Table 7:** Comparison of TSA parameters planned in the protocol of the systematic review with actual TSA parameters values used in the published review across continuous outcomes (paired data)

	Sample size (*n*)	Planned mean (95% CI) /Planned reported *n* (%)	Actual mean (95% CI) /Actual reported *n* (%)	Mean difference (95% CI) /Relative Risk (95%CI)	*p*-value
Variance	23	2 (8.7%)	6 (26.1%)	3.00 (0.78 to 19.06)	0.149
MIREDIF	0	---	---	---	---
Alpha	10	0.048 (0.042 to 0.053)	0.039 (0.028 to 0.049)	-0.0089 (-0.0193 to 0.0015)	0.084
Power^a^	2	0.500 (-3.312 to 4.312)	0.900 (0.900;0.900)	0.4000 (-3.4119 to 4.2119)	0.410
Heterogeneity	23	4 (17.4%)	10 (43.5%)	2.50 (0.99 to 8.11)	0.074

There was no significant difference in any of the parameters. MIREDIF ignored

*MIREDIF* Minimal relevant difference, *n* total number of studies

^a^Mean difference in statistical power is reported as a proportion and should be interpreted in percentage points, representing the absolute difference between planned and applied power in the TSA

For continuous outcomes (*n* = 23 with protocol data compared to *n* = 29 without), none of the comparisons reached statistical significance. However, trends favoured more detailed reporting in systematic reviews with protocol data, particularly for power and heterogeneity (Table 8).

**Table 8:** Comparison of TSA parameters used in systematic reviews having planned TSA parameters in their protocol to systematic reviews without detailed information of TSA parameters in their protocol on continuous outcomes

	Mean (95% CI) in those with protocol information / those with protocol information *n* (%) *n* = 23	Mean (95% CI) in those without protocol information / those without protocol information *n* (%) *n* = 29	Mean difference (95% CI) / Relative Risk (95% CI)	*p*-value
Variance	6 (26.1%)	7 (24.1%)	1.08 (0.40 to 2.84)	0.872
MIREDIF	7 (30.4%)	11 (37.9%)	0.80 (0.34 to 1.71)	0.577
Alpha	0.044 (0.038 to 0.050)	0.049 (0.045 to 0.052)	-0.005 (-0.012 to 0.002)	0.149
Power^a^	12 (52.2%)	22 (75.9%)	0.69 (0.41 to 1.04)	0.097
Heterogeneity	10 (43.5%)	7 (24.1%)	1.80 (0.82 to 4.29)	0.147

There was no significant difference in any of the parameters

*MIREDIF* Minimal relevant difference, *n* total number of studies

^a^Mean difference in statistical power is reported as a proportion and should be interpreted in percentage points, representing the absolute difference between planned and applied power in the TSA

### Reporting and consistency of TSA parameters in systematic reviews with TSA protocols compared to systematic reviews without and meta-analysis reports

#### Dichotomous outcomes

Systematic reviews with a TSA protocol (*n* = 112) continued to show superior reporting compared to systematic reviews without TSA protocols and meta-analyses (total *n* = 327). Notably, Pc (RR 0.74; 95% CI 0.63 to 0.86; *p* < 0.001), alpha (mean difference − 0.005; *p* < 0.001), and heterogeneity (RR 0.67; 95% CI 0.54 to 0.83; *p* < 0.001) were significantly more likely to be reported (Table 9).

**Table 9:** Comparison of TSA parameters used in systematic reviews having planned TSA parameters in their protocol to all systematic reviews without detailed information of TSA parameters in their protocol plus meta-analysis reports across dichotomous outcomes

	Mean (95% CI) in those with protocol information / those with protocol information *n* (%) *n* = 112	Mean (95% CI) in those without protocol information / those without protocol information *n* (%) *n* = 327	Mean difference (95% CI) /Relative Risk (95% CI)	*p*-value
Pc^*^	82 (73.2%)	176 (53.8%)	0.74 (0.63 to 0.86)	< 0.001
RRR	0.222 (0.192 to 0.251)	0.889 (-0.416 to 2.195)	0.67 (-0.64 to 1.97)	0.315
Alpha^*^	0.044 (0.042 to 0.046)	0.049 (0.048 to 0.050)	0.005 (0.003 to 0.007)	< 0.001
Power^a^	0.900 (0.896 to 0.904)	0.897 (0.892 to 0.902)	-0.003 (-0.009 to 0.004)	0.424
Heterogeneity^*^	64 (57.1%)	125 (38.2%)	0.67 (0.54 to 0.83)	< 0.001

*Pc* Proportion of participants with the outcome in the control group, *RRR* Relative risk reduction, *n* total number of studies

^a^Mean difference in statistical power is reported as a proportion and should be interpreted in percentage points, representing the absolute difference between planned and applied power in the TSA

^*^Pc, alpha and heterogeneity parameters showed significant differences

#### Continuous outcomes

Across 23 TSA protocol-based systematic reviews compared to 82 systematic reviews/meta-analyses without TSA protocols, no statistically significant differences were observed. However, variance and heterogeneity were reported more frequently in TSA protocol-based reviews than in those without a TSA protocol (26.1% compared to 14.6% and 43.5% compared to 24.4%, respectively) (Table 10).

**Table 10:** Comparison of TSA parameters used in systematic reviews having planned TSA parameters in their protocol to all systematic reviews without detailed information of TSA parameters in their protocol plus meta-analysis reports across continuous outcomes

	Mean (95% CI) in those with protocol information / those with protocol information *n* (%) *n* = 23	Mean (95% CI) in those without protocol information / those without protocol information *n* (%) *n* = 82	Mean difference (95% CI)/ Relative Risk (95% CI)	*p*-value
Variance	6 (26.1%)	12 (14.6%)	0.56 (0.24 to 1.46)	0.190
MIREDIF	7 (30.4%)	23 (28.0%)	0.92 (0.48 to 2.07)	0.821
Alpha	0.044 (0.038 to 0.050)	0.048 (0.047 to 0.050)	0.004 (-0.001 to 0.011)	0.127
Power^a^	12 (52.2%)	56 (68.3%)	1.31 (0.91 to 2.15)	0.207
Heterogeneity	10 (43.5%)	20 (24.4%)	0.56 (0.31 to 1.08)	0.060

There was no significant difference in any of the parameters

*MIREDIF* Minimal relevant difference, *n* total number of studies

^a^Mean difference in statistical power is reported as a proportion and should be interpreted in percentage points, representing the absolute difference between planned and applied power in the TSA

## Discussion

This report provides a comprehensive evaluation of how TSA parameters are reported and adhered to in systematic reviews with either dichotomous or continuous outcomes. We observed inconsistencies between parameters reported in the protocols compared with those reported in the published systematic reviews, reaching significant increases regarding power and heterogeneity in the published systematic reviews. Such increases would lead to increases in the diversity-adjusted required information size and make the TSA more conservative. These inconsistencies reflect two distinct problems, incomplete reporting, which limits transparency, and analytical deviations, which may alter the statistical validity of conclusions. We also noted that having a protocol increased the probability that parameters were reported. Moreover, we observed significant differences between the TSA parameters used comparing systematic reviews with TSA protocols to systematic reviews without TSA protocols and to meta-analysis reports. Collectively, these findings raise important concerns regarding methodological transparency, reproducibility, and research efficiency in the context of TSA use in evidence syntheses.

Among systematic reviews with dichotomous outcomes, we found significant discrepancies between TSA parameters pre-specified in protocols and those ultimately applied in published systematic reviews. Notably, the largest inconsistency was observed for statistical power, where the actual power used in systematic reviews was significantly higher than originally planned. This likely reflects unplanned analytical adjustments, which can lead to overstated precision or false positive results. A significant divergence was also found in the handling of heterogeneity. While proportion of participants with the outcome in the control groups, relative risk reduction, and alpha values were generally reported in both stages, the consistency between planned and actual values remained limited, with only around one-third of studies demonstrating alignment. This discrepancy may reflect post hoc adjustments or a lack of stringent adherence to pre-specified analysis plans. Our findings reiterates concerns raised by Wetterslev and colleagues, who emphasised that misapplication of TSA or failure to adhere to its assumptions can lead to misleading conclusions due to insufficient information size and early false positives [15].

When comparing systematic reviews with TSA protocols to those without, systematic reviews with pre-planned TSA parameters consistently demonstrated better reporting and methodological clarity. Statistically significant differences were noted for proportion of participants with the outcome in the control groups, alpha, and heterogeneity. These patterns persisted when the comparator group was expanded to include also the meta-analysis reports. The advantage of planning TSA parameters in advance appears to be robust across datasets. These findings echo the concerns we raised in 2024 [16], reporting that major TSA parameters were frequently missing in over 50% of systematic reviews and meta-analyses, with transparency rated as poor or very poor in the majority of cases [16]. Furthermore, the Cochrane Handbook (2023) explicitly recommends prospective planning and full justification of TSA parameters in protocols to ensure interpretability and reduce bias [13]. This request has been reiterated in the international research literature whenever frequentist statistical tests are used more than once on accumulating data since 1974 [27].

For systematic reviews with continuous outcomes, the quality of TSA parameter reporting was even poorer. Consistency was extremely low between values in protocols and systematic review, with parameters such as MIREDIF and variance almost entirely absent from protocols. In the paired analyses, no statistically significant differences emerged between planned and actual values, likely due to small sample sizes. In unpaired comparisons, trends suggested that protocol-based reviews reported variance, power, and heterogeneity more frequently than those without protocol information, although these differences did not reach statistical significance. These findings are consistent with those of Xing et al., who showed that only 13% of TSAs were fully reproducible and that continuous outcomes particularly suffered from underreporting of variance and MIREDIF [28]. Due to the limited sample size, caution is advised in interpreting these findings.

Our present results expand upon our findings from the METSA study, which also documented widespread deficiencies in the protocolisation and transparency of TSA parameters [16]. Both our current and previous findings confirm that over half of TSA parameters required for interpreting the validity of results in (Tables 1 and 2), are missing or inconsistently reported [16]. Based on our present study, we conclude that such omissions threaten the integrity of TSA-based conclusions and hinder reproducibility. The meta-epidemiological work of Xing et al. [28] further adds to the evidence by demonstrating that even when data are present, methodological choices are frequently undocumented, thereby limiting replication. Together, these results point to a reproducibility challenge in TSA practice across the evaluated literature.

When comparing systematic reviews with TSA protocols to those without, plus or minus the meta-analysis reports (which were totally without protocols) we found only subtle indications that the reporting of the former was slightly better than in the latter regarding TSA parameters. One reason for us not being able to show that the former were clearly superior may be because the majority were protocols only published in PROSPERO [16]. Such protocols often lack sufficient details. Therefore, the correct way to publish systematic reviews are to both register the protocol in PROSERO plus publish a peer reviewed detailed protocol where one’s intentions are detailed according to guidelines from the Cochrane Collaboration [29] and as emphasised in the present study.

Our study has limitations. To begin with, the literature search was last updated in December, 2021, so it may not include the most recent systematic reviews or meta-analyses published afterward. Because methods and reporting standards continue to evolve especially with newer versions of PRISMA and the growing emphasis on study registration our results might not fully capture the latest developments in review methodology. Furthermore, we also recognise that some degree of subjectivity was unavoidable when extracting and classifying information, even though every effort was made to maintain consistency. Moreover, when we observed differences or failures we did not go back to these authors and requested explanations for these differences or failures. Another limitation is the relatively small number of systematic reviews with continuous outcomes, which restricted the statistical power of our analyses and limited our ability to detect meaningful differences across TSA parameters. As a result, these findings should be interpreted with caution. In addition, our analyses depended on what was reported in the original publications, meaning that incomplete or unclear reporting could have affected certain findings. Despite these limitations, we believe our work offers useful insights into how research practices in systematic reviews and meta-analyses are evolving over time.

The observed inconsistencies and retrospective adjustments in TSA parameters support our call for mandatory specification of TSA parameters in systematic review protocols where TSA is intended. Without clearly defined TSA parameters in advance, the credibility of TSA conclusions is compromised. This aligns with guidance from the Cochrane Handbook (2023) and is supported by Mheissen, who emphasised that appropriate TSA use is essential to avoiding spurious findings and reducing research waste [13, 30]. We support the recommendations made by both Xing et al. [28] and Riberholt et al. [16] that a standard set of minimal reporting items should be mandated for all TSA protocols and should include at least all parameters in (Tables 1 and/or 2). Without stronger enforcement, TSA will continue to be underused or misapplied, limiting its potential to safeguard against spurious evidence. Journals owners, editors, reviewers, and protocol registries should collectively enforce these standards. Doing so will reduce analytical flexibility, prevent selective reporting, and help curtail research waste resulting from underpowered or methodologically ambiguous systematic reviews.

## Conclusions

Our findings emphasise the urgent need for improved methodological transparency in TSA use within systematic reviews. Adherence to pre-planned TSA parameters is inconsistent in the assessed literature, especially for continuous outcomes. Further, discrepancies between systematic reviews and their protocols remain widespread. Systematic reviews with pre-specified TSA plans demonstrate better reporting and methodological clarity. To ensure trustworthy and reproducible conclusions, future systematic reviews should include detailed TSA parameters in their protocols. A coordinated effort drawing on the recommendations of the METSA initiative [16] and Xing et al. [28] is essential to establish and implement reporting standards that ensure TSA fulfils its methodological promise. Meta-analysis reports should be abandoned [5, 6, 8].

## Data Availability

All data will be made available on zenodo.org after publication of our results.

## Abbreviations

AMSTAR II: Assessing the methodological quality of systematic reviews II
Pc: Proportion of participants with the outcome in the control group
RRR: Relative risk reduction
MIREDIF: Minimal relevant difference
D^2^: Diversity
